# The influence of human exploration on the microbial community structure and ammonia oxidizing potential of the Su Bentu limestone cave in Sardinia, Italy

**DOI:** 10.1371/journal.pone.0180700

**Published:** 2017-07-12

**Authors:** Stefan Leuko, Kaisa Koskinen, Laura Sanna, Ilenia M. D’Angeli, Jo De Waele, Paolo Marcia, Christine Moissl-Eichinger, Petra Rettberg

**Affiliations:** 1 German Aerospace Center (DLR e.V.), Institute of Aerospace Medicine, Radiation Biology Department, Research Group 'Astrobiology', Linder Höhe, Cologne (Köln), Germany; 2 Medical University of Graz, Section of Infectious Diseases and Tropical Medicine, Department of Internal Medicine, BioTechMed, Krenngasse, Graz, Austria; 3 Institute for Biometeorology, National Research Council of Italy, Sassari, Italy; 4 Italian Institute of Speleology, University of Bologna, Bologna, Italy; 5 Dipartimento di Scienze della Natura e del Territorio, Università di Sassari, Sassari, Italy; Free University of Bozen/Bolzano, ITALY

## Abstract

The bacterial diversity in the Su Bentu Cave in Sardinia was investigated by means of 16S rRNA gene-based analysis. This 15 km long cave, carved in Jurassic limestone, hosts a variety of calcite speleothems, and a long succession of subterranean lakes with mixed granite and carbonate sands. The lower level is occasionally flooded by a rising groundwater level, but with only scarce input of organic remains (leaves and charcoal fragments). On the quiet cave pools there are visible calcite rafts, whereas walls are locally coated with manganese deposits. In the drier upper levels, where organic input is much more subdued, moonmilk—a hydrated calcium-magnesium carbonate speleothem—can be found. Relative humidity approaches 100% and the measured mean annual cave air temperature is 14.8°C. Samples were obtained in 2014 from calcite rafts, moonmilk, manganese oxide deposits and soil (limestone and granite grains). Microclimatic conditions in the cave near the sampling sites, sample properties, physico-chemical parameters of water, and sediment composition were determined. The microbial community of this system is predominately composed of the phyla Proteobacteria, Actinobacteria, Acidobacteria, Nitrospirae, and Firmicutes. Sampling sites near the entrance of the cave and in close proximity of the underground campsite–located 500 meters deep into the cave—revealed the highest diversity as well as the highest number of human associated microorganisms. Two samples obtained in very close proximity of each other near the campsite, indicate that the human impact is localized and is not distributed freely within the system. Analysis of the abundance of bacterial and archaeal *amoA* genes revealed a far greater abundance of archaeal *amoA* genes compared to bacterial representatives. The results of this study highlight that human impact is confined to locations that are utilized as campsites and that exploration leaves little microbial trails. Furthermore, we uncovered a highly specialized microbiome, which is perfectly adapted to survive and thrive in an environment with low nutrient availability.

## Introduction

Microorganisms inhabit a diverse number of extreme environments such as hot springs, glacial lakes and subterranean systems [1–4]. Due to their subsurface nature, being hosted deep underground, and to the lack of sunlight, caves are nutrient depleted environments where the levels of available organic carbon to support heterotrophic microbial growth are significantly lower than in terrestrial surface ecosystems [5]. As such, underground systems provide a window for analyzing the metabolic potential and flexibility of microbial communities in an aphotic, oligotrophic habitat with potential similarities to diverse globally dominant terrestrial and marine environments [6].

As most caves are formed in carbonated rocks, the majority of microbiological investigations carried out in caves have been described in such systems [7–12]. Some studies have also been done in quartzite caves in Venezuela, which are characterized by an even lower mineral diversity [13]. Similar to extreme conditions on the surface, microbial communities have adapted to oligotrophy in subterranean environments; despite these low nutrient conditions, the average number of microorganisms thriving in these subterranean systems is estimated at 10^6^ cells/g of rock [14]. Studies of the microbial composition prevalent in oligotrophic cave settings revealed a surprisingly high degree of diversity within the domains of Bacteria and Archaea [2]. Representatives of the phylum Proteobacteria are prevalent and abundant in caves such as the Tito Bustillo cave in Spain [15] or the karstic Herrenberg cave in Germany [16]. Furthermore, this group represents the dominant phylum in biofilms, matrix-enclosed bacterial populations adherent to each other and the surface [17], which have been studied in serval other caves such as the Grotta de Fiume ([8]), karst systems in Slovenia [18] and the Lower Kane Cave in Wyoming [19].

Nitrification, the aerobic oxidation of ammonia to nitrate via nitrite, has been suggested to play a critical role in the global biogeochemical nitrogen cycle since the oxygenation of Earth [20]. For closed or semiclosed environments, such as caves, nitrogen fixation by Bacteria and Archaea can be the main source of bioavailable nitrogen for other organisms while ammonia from organic matter mineralization and /or guano deposits can be a source of energy for chemolithotrophic organisms [21]. In recent years, the newly discovered Thaumarchaeota have gathered a lot of attention in terms of their ability to thrive in environments with low nutrient availability [6, 22, 23]. Other studies have identified Thaumarchaeota as chemoautotrophic ammonia-oxidizers and have shown them to fix CO_2_ using the 3-hydroxypropionate/4-hydroxybutyrate (HP/HB) cycle [24]. They have been detected by molecular surveys in many different environments, including the water column [25], soils [26], freshwater sediments [27] and subterranean habitats [13, 28].

The impact of human interference on the microbial composition of subterranean systems has been an issue for many decades. Several karst systems are open to the public, e.g. the Naracoorte cave in Australia or the Lascaux Cave in France, and the anthropogenic influence of frequently visiting such environments has been often investigated [29, 30] Other caves have limited access, e.g. the Lechuguilla Cave in the USA, which has been closed for free human visits 20 years ago [10].

For this study we investigated the microbial diversity of the Su Bentu Cave, a karst system located in the north-western part of Supramonte karst massif (Central-East Sardinia, Italy). The cave comprises over 15 km explored passages and most of the cave is not subjected to flooding, creating a typical oligotrophic environment. This cavity is not a show cave; however, several local and international speleologists frequent this system throughout the year. Within the cave, one spot is regularly used as campsite (described in detail in Material & Methods), which serves as a test-bed to investigate the human impact of short-term settlement on the present microbial diversity.

Besides the naturally occurring biosphere, this study also focuses on the potential human impact occurrence on the microbiome within this subterranean environment and the presence, abundance and distribution of ammonia-oxidizing bacteria and archaea. To deepen our understanding of the microbial interactions within subterranean environments, we elucidated the microbial diversity, as well as the impact of human exploration on the native system of the Su Bentu Cave in Sardinia, Italy.

## Material and methods

### The Su Bentu Cave

Su Bentu Cave is located in the Supramonte karst massif (Central-East Sardinia, Italy), a Mesozoic carbonate plateau, 9 km wide and 20 km long corresponding to an area of 170 km^2^, topographically elevated over a Paleozoic crystalline basement. With its biodiversity, this region of mountain wilderness preserves small areas of oak and juniper forest surrounded by wide degraded shrubland. This plant community is constituted by sclerophyll species over a very thin soil and by mountain garigue on bare rock pavement [31]The climate is semi-arid with an annual mean temperature and precipitation of 13°C and 1.100 mm, respectively. Rain mainly falls during spring and autumn, separated by a long summer drought [32]. The Su Bentu underground network is hosted within Jurassic and Cretaceous limestones along a large reverse faults system that delineates to the west, and an east-facing monocline slope constituted by the flank of the Tertiary syncline of Lanaitto Valley [33]. With its three entrances, one of them connected to Sa Oche sump, Su Bentu Cave opens at 206 m above sea level (asl) in the southern margin of this geological structure. It comprises more than 15 km of explored cave passages, for a vertical range of 210 meters developed between approximately 105 meters and 315 meters asl. The karst conduits expand almost horizontally over an area of about 6 km^2^ and more than 200 meters underneath the surface. They are organized in two main branches: the Lakes Branch, a seasonally flooded underground canyon where an ephemeral stream fed by the phreatic passages at 105 meters asl flows at a lower cave level, and the 4^th^ Wind Branch, a network of dry looping tunnels of considerable dimensions interconnected through large chambers where dripping water creates emerald pools in well-decorate passages. Both branches converge close to the narrow passage called 4^th^ Wind, at a distance of 1,000 meters from the entrance, creating the huge room of Sala Piredda and the “Grande Cengia”. The whole cave system is connected to Su Gologone spring, the major of Supramonte aquifer, located at 104 m asl in the northern edge of the karst massif. Surface runoff in the area is typically absent. The present day recharge of the cave tributaries occurs predominantly during rainfall events by direct infiltration and/or by the rising water table. Its hydrological catchment comprises carbonate rocks, mainly limestones, while there is strong evidence of past allogenic water input from the Paleozoic basement, now at a lower elevation, constituted by weathered granite and metamorphic rocks [34].

### Sampling sites

**Fig 1:**
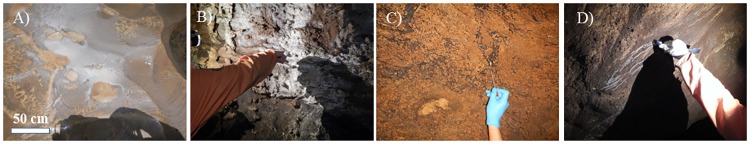
Representative sampling sites in the cave. A) Calcite raft deposit at Chaos (SO1); B) moonmilk deposit at Piredda Hall (SO2 & SO3); C) manganese oxide deposit at Water Tower site (SO6) and D) sampling of a limestone wall at location Shaft (SO4)

The samples used in this analysis were collected in 2014 at 5 different remote areas of Su Bentu Cave, namely Ball Room, Shaft, and Water Tower, that only occasionally have been impacted by caver activity, as well as Piredda Hall which is close to a frequently used campsite during explorations and Chaos, a site close to the entrance were all explores have to pass to get into the cave. At these locations the following samples were taken: loose, solid calcite raft samples from “Chaos” (SO1), moonmilk samples from “Piredda Hall” (SO2 and SO3) by aseptically scraping material in a 50 mL falcon tube (SO2) and an additional sample 5 cm adjacent to the first sample by swiping a flocked swap over the approx. 1 cm^3^of moonmilk (SO3). The limestone cave wall was scraped off at the location “Shaft” (SO4), two samples from manganese oxide were taken at “Water Tower”, one was scraped into a 50 mL falcon tube (S05), the other sample adjacent by swiping a flocked swap over the approx. 1 cm^3^of the manganese deposit (SO6) and a sediment sample was taken from “Ball Room” (SO7). Representative sampling sites are shown in Fig 1 and are described in detail below.

Chaos (SO1) is the most downstream part of the lower Lake’s level and connects through a sump with the Sa Oche cave entrance. It can be accessed descending a series of ropes down to 40 meters below the Witch’s Hat conduit and the main upper cave level. The sample was taken close to the sump area, approximately 5 meters above water level at the time of sampling. The sample regarded the calcite rafts, which were lying on the rocky floor of the cave together with brown muddy sediments. These calcite rafts originally were floating flakes of calcium carbonate formed by oversaturation of the standing water body caused by evaporation of the water. The lowering of the water level caused these rafts to be abandoned on the cave floor. The fine sediments that accompany these white rafts are residual clays mostly of local origin (insoluble remains of limestone dissolution).

Piredda Hall (SO2 and SO3) is a very wide cave room located at only less than 100 meters from the main campsite in the cave. This hall forms the junction between the Lakes Branch and the 4^th^ Wind branch, and is characterized by the confluence of the air masses flowing though these separate branches. The mixing of these air masses causes formation of underground clouds, where condensation occurs on the upper parts of the cave voids, while evaporation predominates in its lower parts. The over 20 meter high cave roof is characterized by cupola-like and ceiling channel-like morphologies, and lacks any type of speleothem. The lower part hosts flowstones, gours, and some other vadose speleothems. The microbiological samples have been taken along the walls of the cave where white toothpaste-like soft deposits are growing (known as moonmilk). Although this area is frequently visited by cavers, the sampling spot was out of the main path.

The sampling spot “Shaft” (SO4) is located 500 meters deeper into the cave, at the base of a vertical passage, a few meters from Rainbow Lake. This shaft is 15 meters deep and leads into a lower series of galleries that are seasonally flooded by infiltrating waters. Less than 100 meters further into the cave, Baikal Lake is encountered, a small pool that forms a sump during these high water periods. The sampling area is characterized by a floor composed of permanently wet flowstone, together with sands (granite and limestone grains) and some organic material brought in by the infiltration waters.

Sampling site “Water Tower” (SO5 and SO6) is located deep into the cave, at the end of the Lakes series. The cave rooms become very large with a sandy floor and a roof up to 80 meters high at some places. This area is flooded seasonally for a couple of days, and the floor is characterized by an admixture of granite and carbonate sands and some organic material. The Water Tower is situated below an area with intense dripping, and a permanent pool is present on a ledge above. Although not frequently used by cavers, the sampling spot is located on the obligate passage point toward the final rooms of the cave. The sampled material occurs as black coating on the cave walls that can be referred to as manganese oxides, the most common black wall crust in caves [35].

The sampling site “Ball Room” (SO7), located 200 meters from “Water Tower”, is characterized by spherical intrasediment concretions now exposed on the cave floor (the “balls" of the room), and a roof only a few meters high. This part of the cave is seasonally flooded during high flow, the water rising from the nearby shaft. When this happens many features indicate the flow to be rather turbulent, creating vortices that have eroded all loose sediments leaving only these hardened parts (balls) on the floor. At the end of these floods water subsides slowly leaving muddy sediments mixed with some organic material. Samples were collected from the cave floor and consisted of limestone sands and muddy sediments.

### Environmental factors and physico-chemical analysis

**Fig 2:**
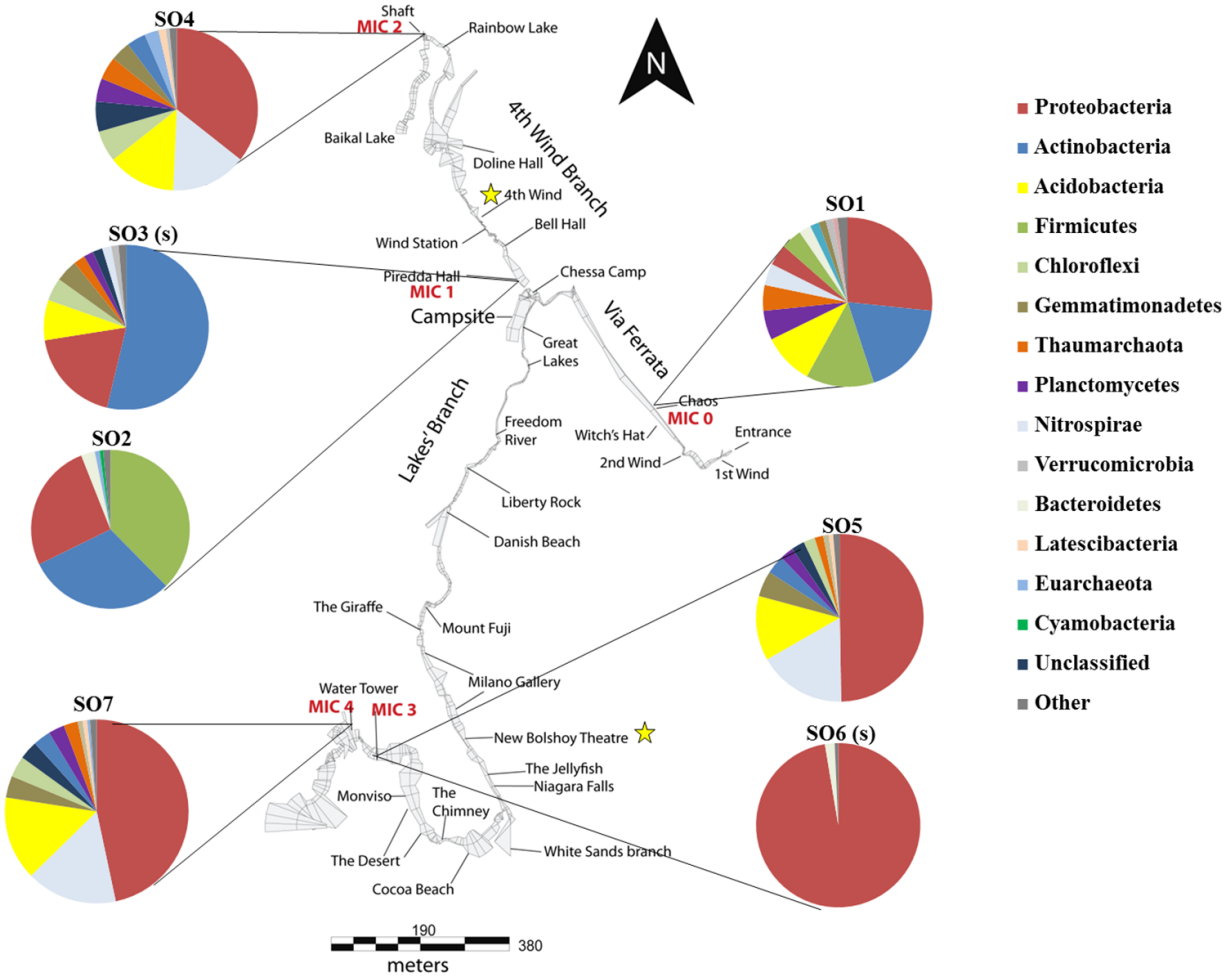
Phyla distribution at different sampling points. Illustrated are the bacterial phyla and proteobacterial classes found within the cave. Taxonomic classification was performed according to SILVA SSU rRNA database 123. Bacterial phyla with a relative abundance lower 0.5% were summarized in the artificial group “Other”. This group includes representatives of the following phyla: Deinococcus-Thermus, JL-ETNP-Z39, Chlorobi, Saccharibacteria, SHA-109, TM6, Armatimonadetes, Chlamydia, Elusimicrobia, WD272, Candidate_division_OP3, Parcubacteria, Fibrobacteres, Fusobacteria, OC31, TA06, WCHB1-60, Aenigmarchaeota, Spirochaetae, Thermotogae, Lentisphaerae, Synergistetes, Candidate_divison _SR1, SM2F11, and Omnitrophica. Two yellow stars indicate representative sampling locations for the chemical analysis of the 4^th^ Wind branch and the Lakes Branch.

As cave microclimate is almost constant [36], the micrometeorological parameters in Su Bentu Cave were measured at 1,200 meters from the entrance and at 30 minutes intervals with three Onset HOBO U23 Pro v2 Temperature/Relative Humidity data loggers (resolution: 0.02°C accuracy: ± 0.21°C for temperature and resolution: 0.03% accuracy: ± 3.5% for relative humidity, respectively). The sensors were placed close to the Piredda Hall sampling point at the confluence between the 4^th^ Wind Branch and Lakes Branch, at three different levels along a 40 meters high vertical profile from the thalweg of the canyon (Great Lake) to the Chessa Camp, and up to the roof of a frequently used campsite (Piredda Hall). Cave air circulation was measured continuously 100 meters from this last place, between Bell Hall and 4^th^ Wind passage (Fig 2 “Wind station”), using a CR200 Campbell Scientific Inc. data logger equipped with a Gill 1 wind sonic anemometer (3° and 2% of wind direction and wind speed accuracy, respectively). Spot measurements of carbon dioxide concentration in the cave atmosphere were performed with a portable NDIR sensor (Zenith AZ7755, range 0–10,000 ppm—accuracy ±50 ppm).

Two aliquots of water were collected in disposable containers with no headspace for chemical analysis, one at Rainbow Lake in the upper 4^th^ Wind branch (a still water pool above and close to sample SO4), the second in a lake below campsite, in the lower Lakes Branch. Two hundred and fifty mL samples were collected for major anions (SO_4_^2-^, Cl^-^, HCO_3_^-^ and NO_3_^2-^) and fundamental metals (Na^+^, K^+^, Mg^2+^ and Ca^2+^) respectively. Samples for cation analyses (100 mL in volume) were filtered with a 0.45 μm sterile filter and acidified with 1 mL of concentrated HNO_3_. The water physical parameters were determined in situ with a portable Hanna HI 991301 probe measuring pH, temperature (T) and electrical conductivity (EC). The range of this probe is between 0.00 and 14.00 for pH (resolution: 0.01; accuracy: ± 0.01), between 0.00 and 20.00 mS/cm for EC (resolution: 0.01 mS/cm; accuracy: ± 2%) 0.00 and 10.00 ppt (g/L) for TDS (resolution: 0.01 ppt; accuracy: ± 2%) and 0.0 to 60.0°C for the temperature (resolution: 0.1°C; accuracy: ± 0.5°C). The alkalinity was also determined in situ as bicarbonate ion concentration (HCO_3_^-^), by titration with methyl orange and hydrochloric acid. The saturation index (SI) of calcite was computed using Merlak’s algorithm [37]. Water samples were analyzed at the University of Bologna (Italy) using an Atomic Absorption Spectrophotometer (AAS) and an Ionic Chromatography within two weeks from sampling.

For chemical analysis, two clastic sediment samples were collected on the cave floors in the 4^th^ Wind Branch and the Lakes Branch (Fig 2, indicated by yellow stars) and are representative for the two main soil types in the cave. There was not enough material to perform detailed chemical analysis on the calcite rafts, moonmilk deposits and manganese oxide. The bulk chemical composition of two sediment samples were obtained by a wave dispersive X-ray fluorescence spectrometer (WD-XRF) (Panalytical Axios, XRF Laboratory, BIGEA–Bologna) on pressed powder pellets, following the matrix correction methods of Franzini et al. [38], Leoni and Saitta [39], and Leoni et al. [40]. Calibration is based on 35 international reference materials and the estimated precision and accuracy for trace-element determinations are better than 5%, except for elements at concertation <10 ppm (10–15%). Volatile content (LOI) was evaluated by thermogravimetric TG-DTG-DTA analysis (XRF Laboratory, BIGEA–Bologna) in air atmosphere using a Setaram Labsys double-furnace apparatus (temperature range 20–1,050°C; heating rate 10°C/min; platinum crucibles; calcined Al_2_O_3_ as reference substance; flow rate of air 0.27 mL/s; temperature accuracy about ±1°C).

### Microbiological sample collection

Seven samples were taken during cave exploration at the previously described five sampling sites: Approximately 5 g of calcite raft, manganese oxide or moonmilk were sampled with a sterile spoon and filled into a 50 mL falcon tube containing 15 mL of *RNAlater* and mixed vigorously. In addition, two flocked swap (MicroRheologics) samples were obtained from moonmilk (SO3) and the manganese oxide deposit (SO6). Samples were stored at ambient temperatures (approx. 14°C) until the end of the expedition and put immediately on ice once the expedition crew left the cave. Samples were transported at 4°C and upon arrival at the laboratory immediately stored at -80°C.

### DNA extraction and quality assessment

DNA from the calcite raft (SO1), moonmilk (SO2), limestone (SO4, SO7) and manganese deposit (SO6) samples was extracted using the PowerSoil extraction kit (MoBIO) according to the manufacturer’s protocol. Briefly, 0.25 g of soil was employed and DNA was extracted using a combination of bead-beading and lysis buffer. DNA was eluted into a final volume of 50 μL dH_2_O. DNA from flocked swap samples (SO3 and SO6) was extracted using the XS-lysis buffer method as described in detail by Tillett and Neilan [41]. DNA was purified with a standard PCI (25:24:1) purification and Isopropanol precipitation followed by to washes with 75% EtOH. DNA was re-suspended in 50 μL dH_2_O. The concentration of extracted DNA was determined with a Nanodrop spectrophotometer at 260 nm. The quality of the extracted DNA was tested by the following PCR setup and protocol. Reactions were performed in 20 μL containing 1 U Platinum *Taq* polymerase, 1 x polymerase buffer, 3 mM MgCl_2_, 0.2 mM dNTP’s, and 0.5 μM of PCR primers 515F (5’GTGCCAGCMGCCGCGGTAA’3) and 806R (5’ GGACTACHVGGGTWTCTAAT’3) [42]. Amplification followed the protocol provided by the Earth Microbiome project website (www.earthmicrobiome.org) and is described there in detail.

### Illumina MiSeq analysis and data processing

The community composition and diversity of Archaea and Bacteria in cave samples were studied using amplicon sequencing method: a variable region of 16S rRNA gene, present in Bacteria and Archaea, were amplified with universal PCR primers 515F (5’GTGCCAGCMG-CCGCGGTAA’3) and 806R (5’ GGACTACHVGGGTWTCTAAT’3) [42]. The produced fragments were subjected to Illumina MiSeq sequencing process. The produced data were analyzed using publicly available algorithms and analysis pipeline, Mothur, following the MiSeq standard operating procedure (SOP) [43] (SOP accessed 23.5.2016). In short, the paired end reads were joined together, and the produced sequences were quality checked. Chimeric sequences were identified and removed, and the sequences were clustered into OTUs using average neighbour algorithm. Taxonomic assignment is performed by querying the sequence reads against a silva SSU 123 reference database [44] and various diversity indices and richness estimates were calculated. Downstream data analysis was performed with Sigma Plot 13.0 and the online software Calypso (http://cgenome.net/calypso/) [45]. Sequence data were deposited in the European Nucleotide Archive (ENA) with the study accession number PRJE19599.

### Abundance of bacterial and archaeal amoA genes

To screen for the presence and amount of bacterial and archaeal *amoA* genes, quantitative PCR (qPCR) was employed. To identify bacterial *amoA* genes, primers amoA-1F (5’ GGGGTTTCTACTGGTGGT ‘3) and amoA-2R (5’ CCCCTCKGSAAAGCCTTCTTC ‘3) were used and genes amplified as previously described by Rotthauwe et al. [46]. To identify archaeal *amoA* genes, primers Crenamo1F (5’ AATGGTCTGGCTWAGACGC ‘3) and CrenAmo1R (5’ GACCARGCGGCCATCCA ‘3) were used and amplified as previously described by Könneke et al. [47]. The qPCR was performed in an Opticon2 system (BioRad) using the PeqLab KAPA Sybr FAST Kit, three replicates of each sample. Melting curve analysis (0.2°C s^-1^) and agarose gel electrophoresis (1% agarose) revealed single amplicons for all samples.

## Results

### Microclimatic conditions

Relative humidity at each site approaches 100% and the measured mean annual cave air temperature is 14.8°C (±0.18°C). The altitudinal thermal gradient between the sampling sites is 0.05°C/m, with values ranging between 16.0°C at Piredda Hall and 13.9°C at the Water Tower sampling site. Air circulation inside the cave follows a diurnal and seasonal pattern showing a change in direction of the movement of air masses depending mostly on outside temperature. Airflow at the “Wind station” (4^th^ Wind Branch, Fig 2) measuring point has a maximum speed of 10 m/s in summer. During winter this speed ranges from 1 to 6 m/s and a temporary sump can be filled with rain closing the passage completely, and stopping the cave ventilation for a couple of days. Air pCO_2_ in cave atmosphere is different at the sampling areas showing values of 4,000 ppm at Piredda Hall in the well-ventilated upper level and 2,680 ppm in the quiet lower level of Ball Room.

### Physico-chemical analysis of water

Two water samples collected during the expedition display the same temperature, a quite similar specific electrical conductivity (EC) and total dissolved solid (TDS), and an almost neutral pH (Table 1). The concentration in Ca^+^ exceeds that of Mg^+^, and the predominant anion is HCO_3_^-^, so both are a calcium-bicarbonate type and have a negative calcite saturation index. Na^+^ and Cl^-^ concentration slightly increases in the Campo Chessa sample with respect to the Lakes Branch one. Of notable exception is the slightly higher level of sulphate in this last site and the slightly higher nitrate content at the Campo Chessa sampling site.

**Table 1:** Water chemical parameters from representative areas in the cave. SI_calcite_ was calculated using the Merlak algorithm [37].

Site	pH	T (°C)	EC (mS/cm)	TDS (ppt)	HCO_3_^-^ (mg/L)	Cl^-^ (mg/L)	NO_3_^-^ (mg/L)	SO_4_^2-^ (mg/L)	Na^+^ (mg/L)	K^+^ (mg/L)	Ca^2+^ (mg/L)	Mg^2+^ (mg/L)	SI_calcite_
4^th^ wind branch	7.6	13.9	0.37	0.18	110.9	11.26	2.07	3.57	8.36	0.31	34.38	1.89	-0.6
Lakes branch	7.3	13.9	0.39	0.19	94.2	10.41	1.99	6.79	6.89	0.33	30.86	1.52	-0.7

### Sediment properties

The bulk chemical analyses of major elements in the sediments of the two main branches (upper and lower) of the cave system is shown in Table 2, expressed as mass fraction (weight percentage, wt%). Even though carbonate forms the essential part of the bedrock, the cave sediments are allogenic fluvial deposits formed outside of the karst system and transported into it by seasonal flow. Angular grains of quartz and feldspar mainly constitute the granite sand at the Lakes Branch site whereas the red clay deposit at the 4^th^ Wind sampling site is dominated by phyllosilicates. It is not surprising that Si ions predominate in the chemical composition. Absolute abundance ranges from 80% for the granite sand to 38% for the muddy clay, respectively. The different silicate minerals are also the major carrier of Al, the second most abundant element. The abundance of Fe is mainly coupled with the occurrence of oxides and hydroxides incorporated in the crystal lattice of clay minerals. The amount of Ca is low compared to most of the cave sediments and the greater part is related to carbonate residues in the clay fraction. The volatile content (LOI) indicates low amount of organic matter in both samples.

**Table 2:** Chemical composition (XRF) of the predominant cave sediments.

Description	SiO_2_ (wt %)	TiO_2_ (wt %)	Al_2_O_3_ (wt %)	Fe_2_O_3_ (wt %)	MnO (wt %)	MgO (wt %)	CaO (wt %)	Na_2_O (wt %)	K_2_O (wt %)	P_2_O_5_ (wt %)	LOI
4^th^ wind branch	38.64	1.15	16.81	8.71	0.24	1.84	10.64	0.15	2.10	0.36	19.36
Lakes branch	80.05	0.20	7.56	2.57	0.04	1.28	2.39	0.51	1.95	0.04	3.42

### Diversity and characteristics of the microbial community

**Fig 3:**
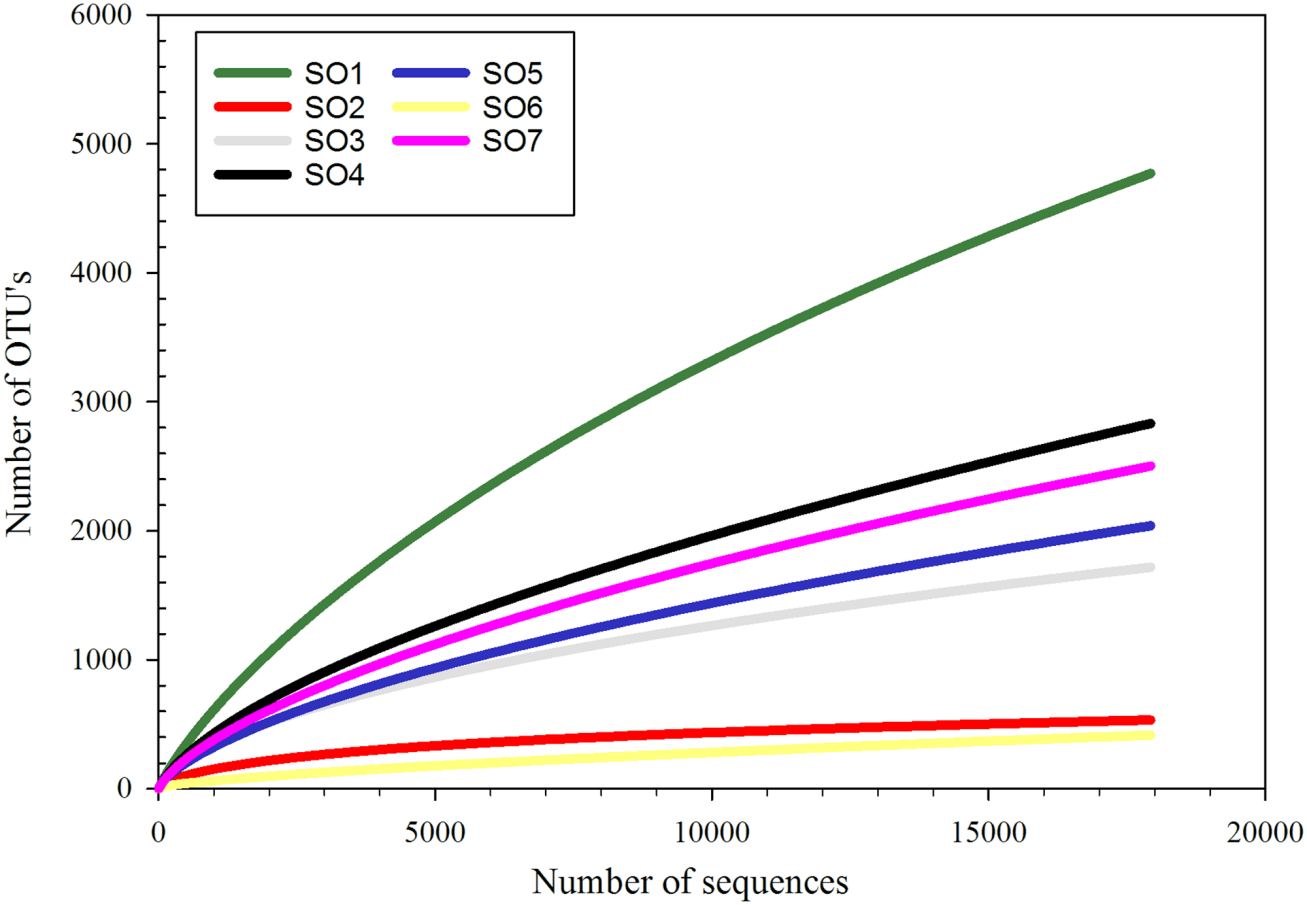
Rarefaction analysis of the microbial communities at the different sampling points. Curves were calculated by MOTHUR with a 3% distance cutoff.

The microbial diversity of the Su Bentu Cave in Sardinia has been investigated at several different sampling locations (Fig 1). Based on the observed OTU’s, SO1 had the highest diversity (4771), while the lowest diversity was observed at SO6 (418) (Table 3). The Inverse Simpson index as well indicates a high diversity at SO1 (140.94) compared to a very low diversity at SO6 (2.88). All other samples fall between these two values (Table 3). Rarefaction curves of alpha diversity observed for all sampling opportunities are shown in Fig 3 and generally indicate near saturation, except for SO1. Coverage was calculated using the Good’s algorithm and is given in Table 3. All sampling efforts showed a coverage >90%, except for SO1 with a coverage of 84.21%, indicating that the majority of microbial phylotypes present were recovered for the other sampling sites, but not for SO1.

**Table 3:** Bacterial and archaeal 16S rRNA gene diversity analyses of samples obtained from the Su Bentu Cave in Sardinia.

Sampling location	Abbreviation	No. high quality reads	No. OTU	Coverage (%)	Inv. Simpson
Chaos	SO1	15.651	4771	84.21	140.94
Piredda Hall (soil)	SO2	17.781	534	99.01	14.79
Piredda Hall (swap)	SO3	17.249	1718	95.14	99.15
Shaft	SO4	16.541	2833	90.21	99.83
Water Tower (soil)	SO5	17.096	2040	93.35	41.18
Water Tower (swap)	SO6	17.714	418	98.42	2.88
Ball Room	SO7	16.848	2503	91.64	55.81

**Fig 4:**
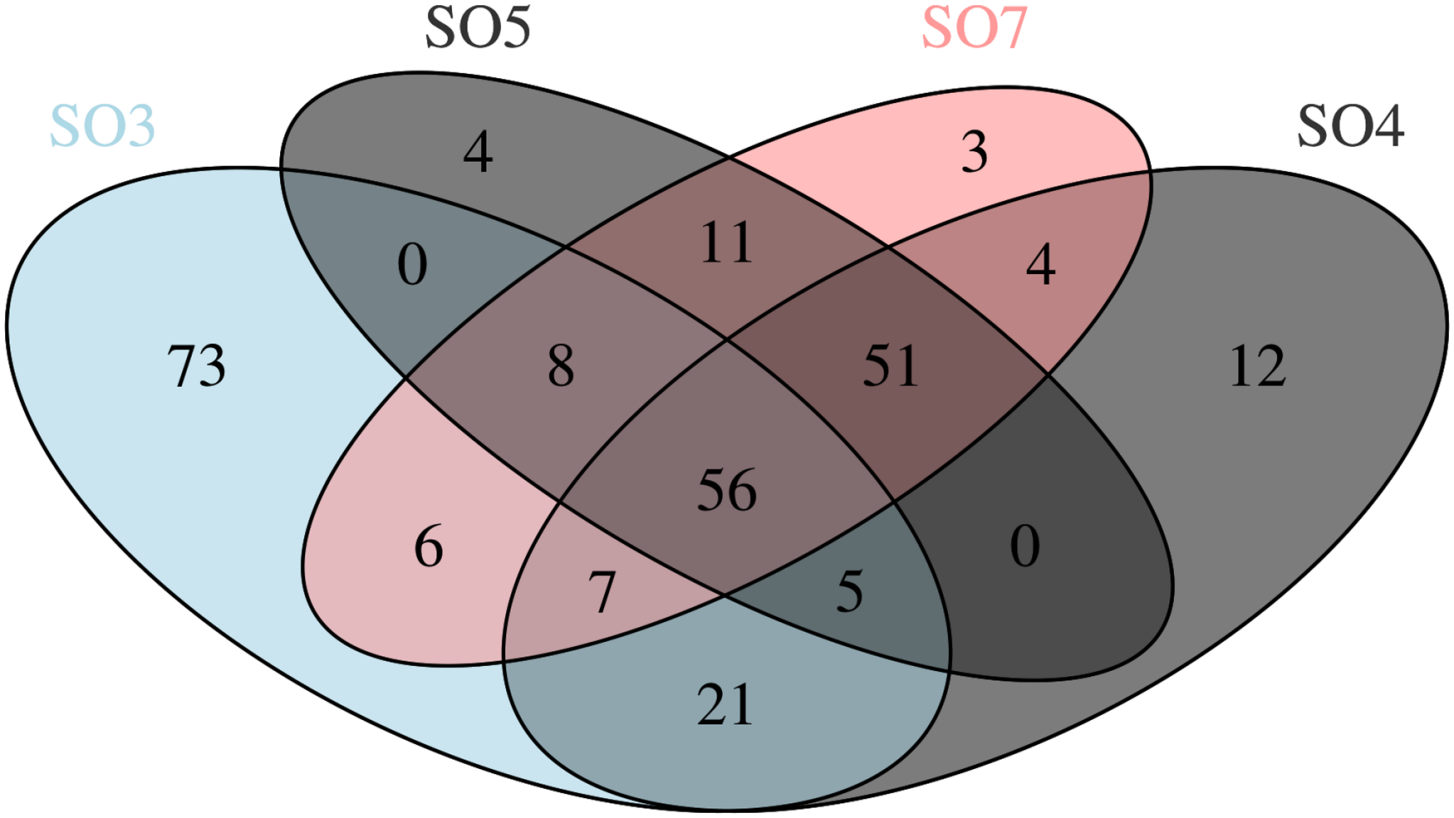
Venn diagram showing the number of core, unique and shared species among the different sampling points. Due to the presence of human contaminations, SO1 and SO2 have been omitted. SO6 has been omitted due to the almost sole presence of Proteobacteria.

Taken together, a total of 37 bacterial and 3 archaeal phyla signatures have been recovered from the different sampling sites. Results indicate that the microbial community (brackets state the total amount of signatures recovered within the whole system) of this cave is predominately composed of the phyla Proteobacteria (43.39%), Actinobacteria (16.21%), Acidobacteria (8.25%), Nitrospirae (7.62%), Firmicutes (7.46%), Chloroflexi (2.91%), Gemmatimonadetes (2.65%), unclassified (2.49%), Planctomycetes (2.45%), and Thaumarchaeota (2.20%) (Fig 2). Notably, sampling point SO6 (a manganese oxide deposit deep inside the cave) was almost exclusively inhabited by Proteobacteria signatures (Fig 2). A complete list of recovered phyla is given in S1 Table. The dominance of Proteobacteria is also apparent when looking at the core microbiome of this environment (Fig 4). SO1, SO2 and SO6 have been omitted from the core microbiome analysis; SO1 and SO2 due to high human impact and SO6 because of the overwhelming presence of Proteobacteria. The Proteobacteria account for 47.27% of recovered phyla, followed by Nitrospirae with 29.76% and Actinobacteria completing the three most recovered phyla with 8.75%. A complete list of recovered core OTU’s can be found in S2 Table.

**Fig 5:**
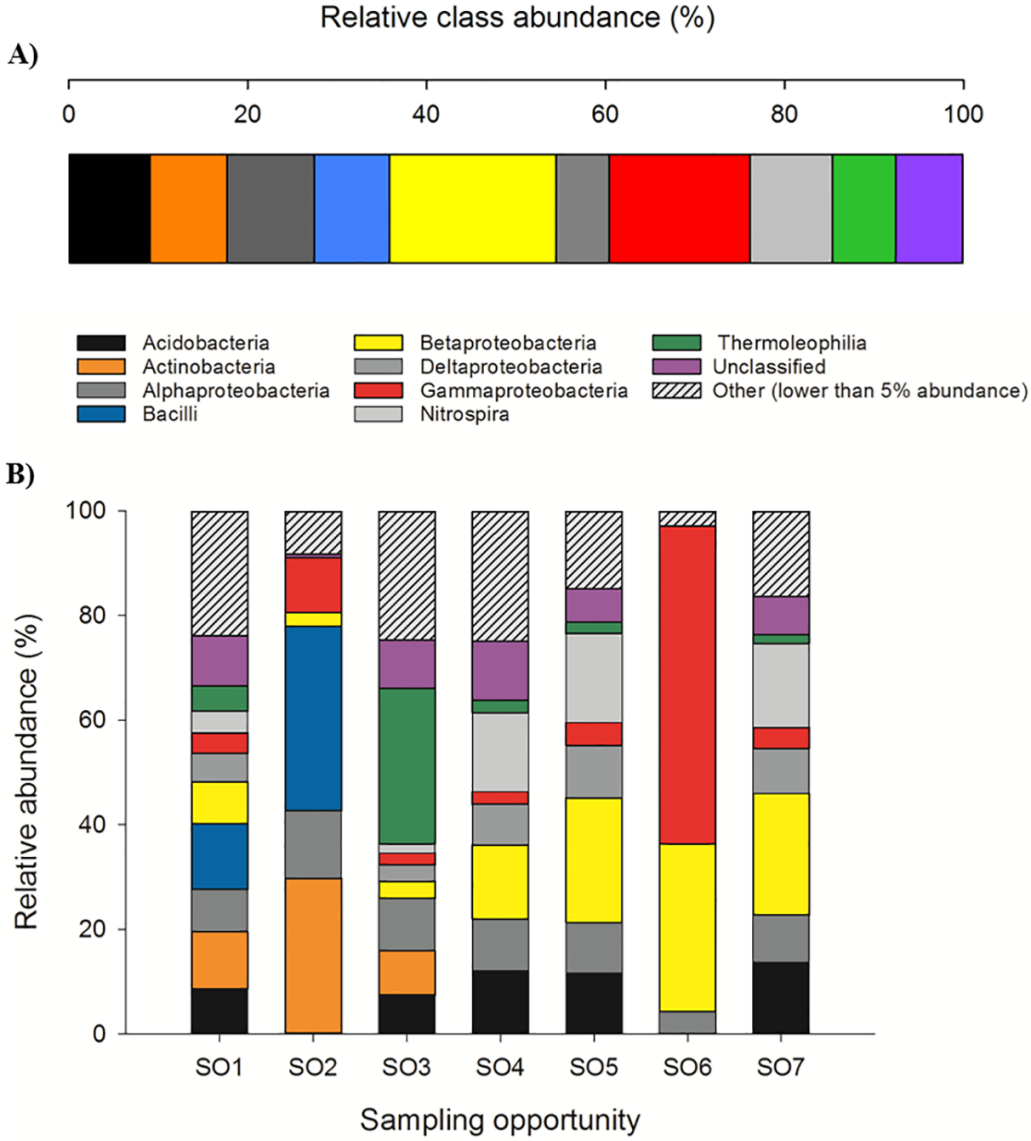
Relative class abundance of A) the whole cave and B) separated into the different sampling sites. Phylogenetic groups accounting for ≤ 5% of all classified sequences are summarized in the artificial group “others” (Part B). Full relative class abundance is given in Supplementary Table 3.

The discovered phyla can be further refined at the class level into β –Proteobacteria (15.38%) being the predominant class of these phyla. This class is followed by γ –Proteobacteria (13.01%), α –Proteobacteria (9.13%), Nitrospira (7.62%), Acidobacteria (7.54%), Actinobacteria (7.15%), Bacilli (7.06%), unclassified (6.30%) and the Thermoleophilia (5.87%) (Fig 5). A complete figure of all classes recovered is given in S1 Fig.

**Fig 6:**
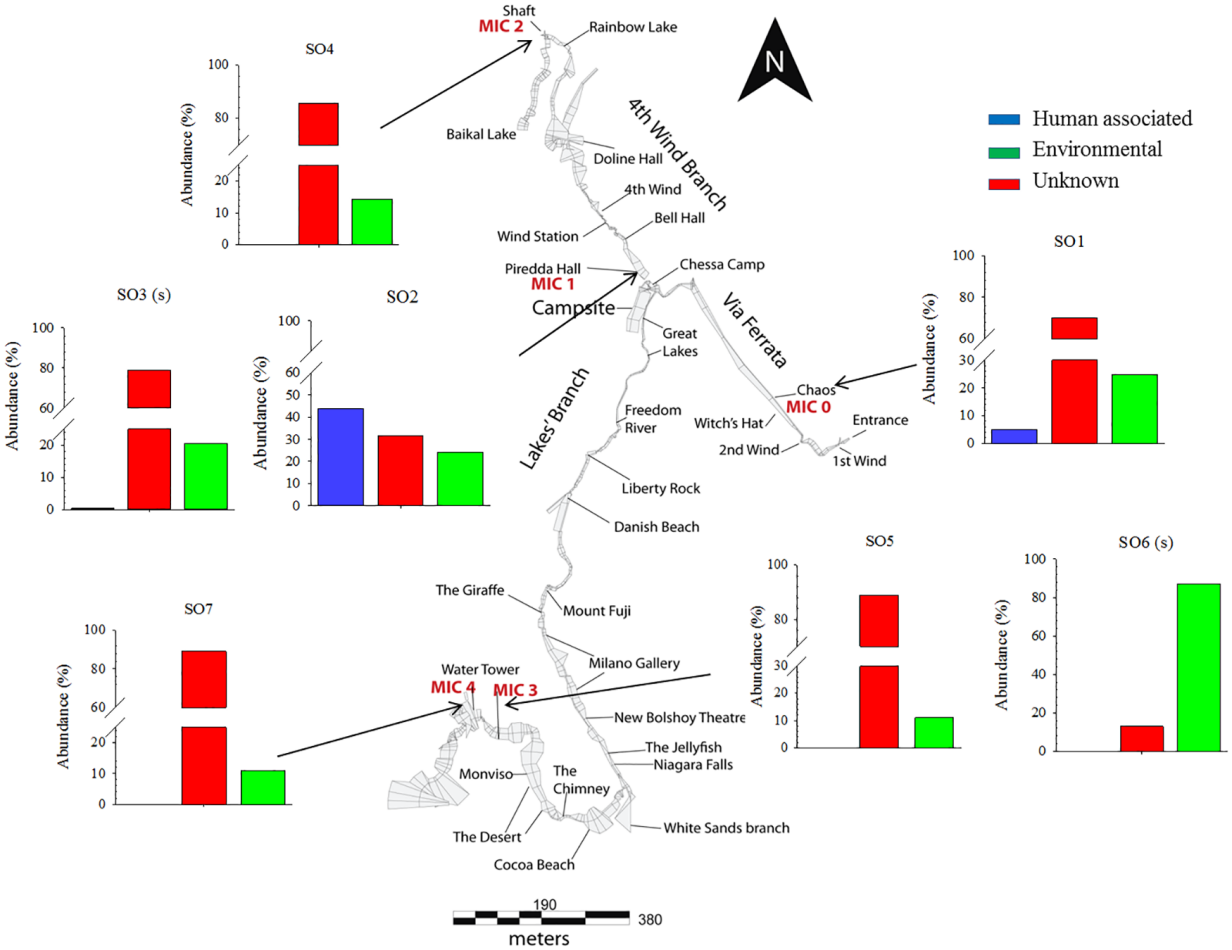
Impact of human exploration on the microbial diversity in a hypogean system. The most abundant human associated genera (as previously described by The Human Microbiome Project Consortium) were used for this analysis and include *Lactobacillus*, *Propionibacterium*, *Streptococcus*, *Bacteroides*, *Corynebacterium*, *Staphylococcus*, *Moraxella*, *Haemophilus*, *Prevotella*, and *Veillonella*.

The presence of signatures of human associated microorganisms has been investigated and results are given in Fig 6. According to research conducted by the Human Microbiome Project Consortium in 2013 [48], we considered the following genera to be of human origin: *Lactobacillus*, *Propionibacterium*, *Streptococcus*, *Bacteroides*, *Corynebacterium*, *Staphylococcus*, *Moraxella*, *Haemophilus*, *Prevotella*, and *Veillonella*. The highest amount of human associated microorganisms has been observed in a moonmilk sample (SO2 with 43.93%) obtained near the campsite followed by the sample obtained from a calcite raft near the entrance of the cave (SO1 with 5%). All other sampling points showed a negligible presence (<0.5%) of human associated organisms (Fig 6). A complete list and distribution of all recovered and identified genera is given in S3 Table.

**Fig 7:**
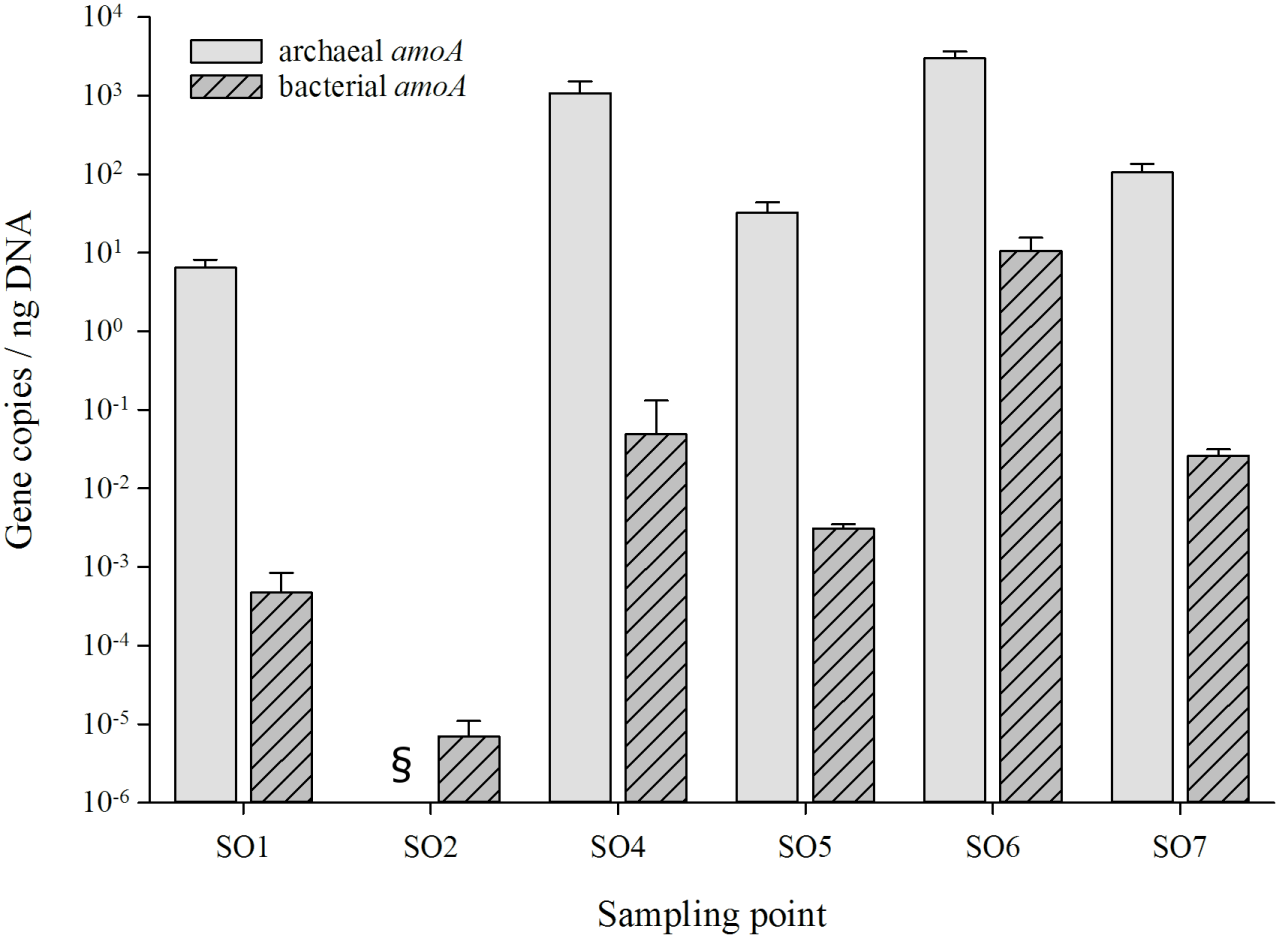
Abundance of the bacterial and archaeal *amoA* genes. § indicates that no amplification was observed at this sampling point for archaeal *amoA* genes. SO3 was not investigated due to the lack of DNA. Standard deviation is given as Error Bars (n = 3).

To investigate the presence/abundance of bacterial and archaeal *amoA* genes within the samples, qPCR analysis was conducted and results are presented in Fig 7. The amount of ammonia (NH_4_^+^) was determined as <0.2 mM for all sampling points. Results indicate that the archaeal ammonia-oxidizing gene is significantly more abundant in most of the samples compared to their bacterial counterpart. Low amounts of *amoA* genes were recovered at SO2, with only bacterial *amoA* genes present. Due to low amounts of DNA recovered at sampling spot SO3, no analysis of the bacterial and archaeal *amoA* gene distribution was possible.

## Discussion

In this study we investigated the microbiome of the Su Bentu Cave, Sardinia by Illumina MiSeq analysis, focusing on the impact of human exploration on the indigenous microbial community and on the ammonia-oxidizing potential as an energy source.

### Geochemical & environmental parameters

Subterranean ecosystems are distinguished by a variety of physical (drip rate, temperature, relative humidity, carbon dioxide partial pressure, water conductivity, and pH) and chemical (chloride, nitrate, sulfate, sodium, potassium, calcium, and magnesium) parameters. They explain the greatest variance of the chemoautotrophic microorganisms that colonize the subterranean realm [49]. The Su Bentu Cave ecosystem is characterized by high relative humidity and a constant air temperature with a limited range of variation, typical of most cave systems [50]. Its intense air circulation is a key microclimatic feature, as its Sardinian language name indicates (“Su Bentu” means “The Wind”). Cave ventilation regulates gas exchanges between underground atmosphere and its surrounding environment (mainly host rock and soil) [51]. Over a short time period investigated, a distinct value of air pCO_2_ in cave atmosphere is evident in the two main cave branches, with lowest values observed in the lower Lakes’ Branch and an increasing concentration in the well-ventilated upper level of Bell Hall room in the 4^th^ Wind Branch, an opposite dynamic respect of the common cave atmosphere with higher CO_2_ values restricted to places with scarce air circulation [52]. Water samples collected during the expedition show no significant differences in the physico-chemical factors of the two different sampling sites. Data related to the physical parameters point out that both waters are poorly mineralized and reflect cave lithology. Hydrochemical content also suggests that in both sites water is typically below saturation with respect to calcite. This means that the moonmilk precipitation is driven by evaporation due to air ventilation. Na^+^ and Cl^-^ concentrations slightly increase in the Campo Chessa area with respect to the Lakes Branch as a consequence of active rainwater infiltration observed throughout the year. Moreover, the more likely sulphate source for the Lakes Branch’s sediment is the oxidation of sulfur minerals (pyrite ores) hosted within granite clasts. Finally, the nitrate contamination is in the range of Sardinian precipitations and reaches the groundwater system as seeping water [53]. The Su Bentu Cave is an oligotrophic ecosystems clearly limited not only by the input of carbon, but also by the availability of other inorganic elements (especially nitrogen and sulfur). The physical parameters dictate the dynamics of these elements that have strong influence on the magnitude and location of the subsurface microbial communities [54].

### Core microbiome

The core microbiome of this environment was assembled from data obtained from SO3, SO4, SO5, and SO7. SO1 and SO2 have been excluded due to the detectable level of human interference and SO6 due to the almost sole presence of the phylum Proteobacteria (Fig 4), discussed later in detail. Signatures of the phylum Proteobacteria are dominant (47.27%) with signatures of the phylum Nitrospira next most abundant (29.76%). Microorganisms from the phylum Proteobacteria are frequently recovered from other subterranean environments such as the Llonin and La Garma caves [55] the Niu Cave [56], the Frasassi cave system [57], as well as on basalt walls of lava caves [58]. Within this phylum the classes of α, β, and γ –Proteobacteria were the most abundant sequences recovered, with β –Proteobacteria being the dominant class (38.99%). Bacteria belonging to the β –Proteobacteria are obligate aerobes and facultative anaerobes, chemoorganotrophs, as well as obligatory or facultative chemolithotrophs [2]. A broad abundance of Nitrospira has been recovered in several other caves such as the Jinjia Cave in China [59], Lechuguilla Cave [60] and Pajsarjeva jama in Slovenia [18]. Nitrospira are capable of autotrophic C fixation [61] and are involved in the two-step autotrophic nitrification, suggesting the presence of the CO_2_ fixation coupled ammonia oxidation process, which likely is the source of primary production in other cave systems [59, 62]. The third phylum, Actinobacteria, is also frequently observed in cave systems where they are known to be involved in the formation of various types of speleothems [12]. Therefore, it is believed that representatives of this phylum are involved in biomineralization processes in their environment [63]. While dominant in other caves such as the Carlsbad Cavern where 80% of recovered population clustered with the phyla Actinobacteria [64], in the Su Bentu cave this phyla seems to play a subordinate role. To end, Acidobacteria were found to be present in moderate abundance (7.02% of core microbiome), with subgroup 6 dominant deep inside the cave (SO4, SO5, and SO7) and subgroup 4 being foremost at SO3. Acidobacterial sequences are commonly found in subterranean environments [65, 66]; however, their role within the ecosystem remains unclear.

### Calcite rafts (SO1)

The precipitation of CaCO_3_ in cave pools occurs because such waters become saturated with respect to CaCO_3_ due to the loss of CO_2_ and evaporation at the air-water interface [67, 68]. By this mechanism, thin crusts of calcite may grow from the walls of the pool across the surface or may form floating calcite rafts [67]. The calcite rafts near the entrance of the cave show the highest microbial diversity recovered from this environment, which is not surprising given the annual flooding and therefore organic input at this site. Furthermore, the close proximity to the entrance where every explorer has to pass increases the chance of external organic input as well as the introduction of allochthonous organisms from the soil outside the cave (e.g. carried in the cave on the shoe of a caver). It is well established that bacteria make a significant contribution to the accumulation of carbonate in the environment [69–71]. Both autotrophic and heterotrophic bacteria, including sulphur and nitrogen-fixing bacteria are involved in CaCO_3_ precipitation [69]. In particular, representatives of the family Bacillaceae have been shown to be actively involved in calcium carbonate precipitation [72, 73], which have been recovered in high numbers at this sampling location.

### Moonmilk (SO2 & SO3)

Moonmilk refers to a variety of microcrystalline mineral aggregates ranging from soft and wet to a dry and powdery appearance [74]. In contrast to moonmilk analyzed in the Altamira Cave in Spain, where α-Proteobacteria were the major components [74], moonmilk recovered from the Su Bentu Cave is dominated by the class of Thermoleophilia (Fig 5), with the dominant order Solirubrobacterales. Members of the Thermoleophilia have been previously isolated from extreme oligotrophic environments such as the Atacama Desert [75]; however, a further classification is not provided. Currently all strains within the order Solirubrobacterales are described as mesophilic and sometimes psychrotolerant [76], which may be part of the explanation on to why members of this order were recovered within a cave with an average temperature of ~14°C.

### Limestone walls & soil (SO4 & SO7)

The limestone samples obtained from this environment appear fairly similar with respect to their relative phyla and class abundance (see Figs 2 and 5), even though both sites are not in close proximity to each other. Similar to other caves [55, 62], Proteobacteria were again identified as the dominant phylum, with β and δ –Proteobacteria the most abundant representatives at these two sites. Furthermore, a high number of Nitrospirales (with genus *Nitrospira* dominant) was recovered from those two sampling sites. Nitrification has long been considered to be a two-step process catalyzed by chemolithoautotrophic microorganisms oxidizing either nitrite or ammonia; however, recent work by Daims and colleagues [77] reports the discovery and cultivation of a complete nitrifying “comammox” (complete ammonia oxidizer) bacterium from the genus *Nitrospira*. Although *Nitrospira*-like bacteria grow very slowly, with generation times up to 90 h for *Nitrospira marina* [78], cultivation attempts would be warranted to further elucidate the nitrification process within this environment.

### Manganese oxide(II) deposits (SO5 & SO6)

Sampling point SO6 revealed almost exclusively the presence of β and γ –Proteobacteria, however, SO5, which was just adjacent to SO6, showed a much higher diversity. The genus almost exclusive recovered at SO6 was *Pseudomonas* (S3 Table). Representatives of this genus, such as *Pseudomonas putida* strain MnB1 are known for their ability of Mn(II) oxidation to form manganese oxide [79, 80]. A similar discovery was reported by Carmichael et al. (2013) [81] in ferromanganese deposits in caves of the upper Tennessee river basin, where *Pseudomonas* was, in addition to *Leptothrix* and *Flavobacterium*-related organisms, the most abundant and detectable population. However, samples taken at the same location (SO5), revealed a far broader diversity with similarities to the previously described diversity for ferromanganese deposits in Lechuguilla and Spider caves [60]. SO5 revealed the presence of the genera *Hyphomicrobium*, *Pedomicrobium* and *Nitrospira*, where its representatives are known for their metal oxidizing abilities [60, 82, 83]. The difference between those results may be attributed to the sampling technique. SO6 was taken with a sterile flocked swap, so only the organisms on the surface were sampled, whereas SO5 was obtained by scraping the surface for a few millimeters with a sterile metal spoon, therefore obtaining more material from different layers of the sampling site.

### Human impact (SO1 & SO2)

The issue of human contamination of a pristine cave environment has been of interest since several decades and numerous studies have shown the impact of tourism or exploration [29, 30, 84]. Cavers and tourists reverse the concentration and availability of organic carbon by bringing fibers, lint, hair as well as human-associated microbes into a cave system [84, 85]. The human microbiome project consortium published in 2012 a list of 16S-identified genera associated with healthy humans [48] that were used to distinguish between human associated or natural environments.

We found a high concentration of human associated organisms in the soil in the area near the campsite (Fig 6). *Propionibacterium* was among the most abundant genera recovered from this area, with *Propionibacterium acnes* being a major inhabitant of the adult human skin, where it resides within sebaceous follicles, usually as a harmless commensal bacterium, although it has been implicated in the formation of acne vulgaris [86]. Furthermore, a high abundance of 16S rRNA sequences belonging to the genera *Staphylococcus* and *Streptococcus* were recovered in similar number compared to *Propionibacterium* (Supplement S3 Table), which are both well-established human associated genera with representative species such as *Staphylococcus epidermidis* [87] and *Streptococcus mutants* [88]. Interestingly, swab samples taken from moonmilk (SO3) in the same location showed no detectable signs of human contamination. Microorganisms in caves range from completely invisible to colorful microbial mats [89], however, the same holds true for human impact. It can be clearly seen as feces or hair, but touching a speleothem (by accident or on purpose) may leave an invisible trace of human associated microorganisms. But this also indicates that the human impact is localized and organisms are not, or only sparsely, transported by wind throughout this system. The only other place where human-associated organisms were retrieved was near the entrance (SO1, Fig 6), a passage everybody has to traverse when exploring the cave, but is also seasonally flooded. This is an interesting scenario as it is well established that water flowing into caves may either bring allochthonous material into the cave yet may also help to wash away some human impact due to exploration [89]. Further research is necessary to untangle this delicate environmental interplay between human impact and natural restoring capabilities.

### Ammonia-oxidizing potential

The microbial oxidation of ammonia is a key process in the global cycling of nitrogen. Nowadays we know that two groups of organisms are responsible for ammonia-oxidation, the ammonia-oxidizing bacteria (AOB) and ammonia-oxidizing archaea (AOA). Previous research has uncovered that AOA often outnumber their bacterial counterparts in marine and terrestrial environments [90, 91]. Only recently was it discovered that ammonia oxidizing archaea dominate over bacterial ammonia oxidizers within alkaline cave sediments of the Heshang Cave in China [92] and the same holds true for the here investigated limestone cave (Fig 7). This high abundance of ammonia-oxidizing archaea (AOA) is probably due to the high substrate affinity, which enables them to grow under far lower ammonia concentrations than other organisms [93]. The low availability of ammonia may be the reason for the higher abundance of AOA in this particular environment. Other factors such as moisture and temperature have also been demonstrated to influence the distribution and activity of Thaumarchaeota [20, 94]. In this study we found that areas with a high presence of human associated bacterial 16S rRNA sequences (SO2) show very little to no presence of archaeal or bacterial *amoA* gene sequences. It may be speculated that due to the increase of non-native microbes at this site, the native microbiome was displaced and eliminated by the invasive microbes. By doing so, cells would have been lysed, releasing DNA and other cellular constituents, which in turn can be used by the invasive species as nutrient source as described for extracellular DNA by Vorkapic et al. (2016) [95]. This may explain the low copy numbers recovered at SO2. Although human impact is detectable at SO1 (“Chaos” near the entrance), the copy number of *amoA* genes was comparable to the other investigated sites. This may be explained by the previously made statement that the regular impact on the microbiome by seasonal water flow may be responsible for this discrepancy.

In this study we elucidated the microbiome of the Su Bentu Cave in Sardinia as well as the impact of exploration on the native microbiome. Similar to other investigated subterranean environments, a broad diversity of different microorganisms was recovered with Proteobacteria being the dominant phyla. It can be concluded that infrequent exploration has a diminutive impact on the indigenous microbial population, compared to higher impacts for touristic caves [e.g. 29]. Further in-depth studies will certainly lead to the discovery of novel species with yet unknown traits for survival in low nutrient environments.

## Supporting information

S1 Table Complete list of recovered phyla. (XLSX)

S2 Table The core microbiome of the Su Bentu Cave. (XLSX)

S3 Table Complete list of recovered genera. (XLSX)

S1 Fig Full class abundance of the Su Bentu Cave. (TIF)
