# Supplementary figures and images for: The influence of human exploration on the microbial community structure and ammonia oxidizing potential of the Su Bentu limestone cave in Sardinia, Italy
Source: PLoS One. 2017 Jul 12;12(7):e0180700. doi: 10.1371/journal.pone.0180700 (PMC5507542; doi:10.1371/journal.pone.0180700)

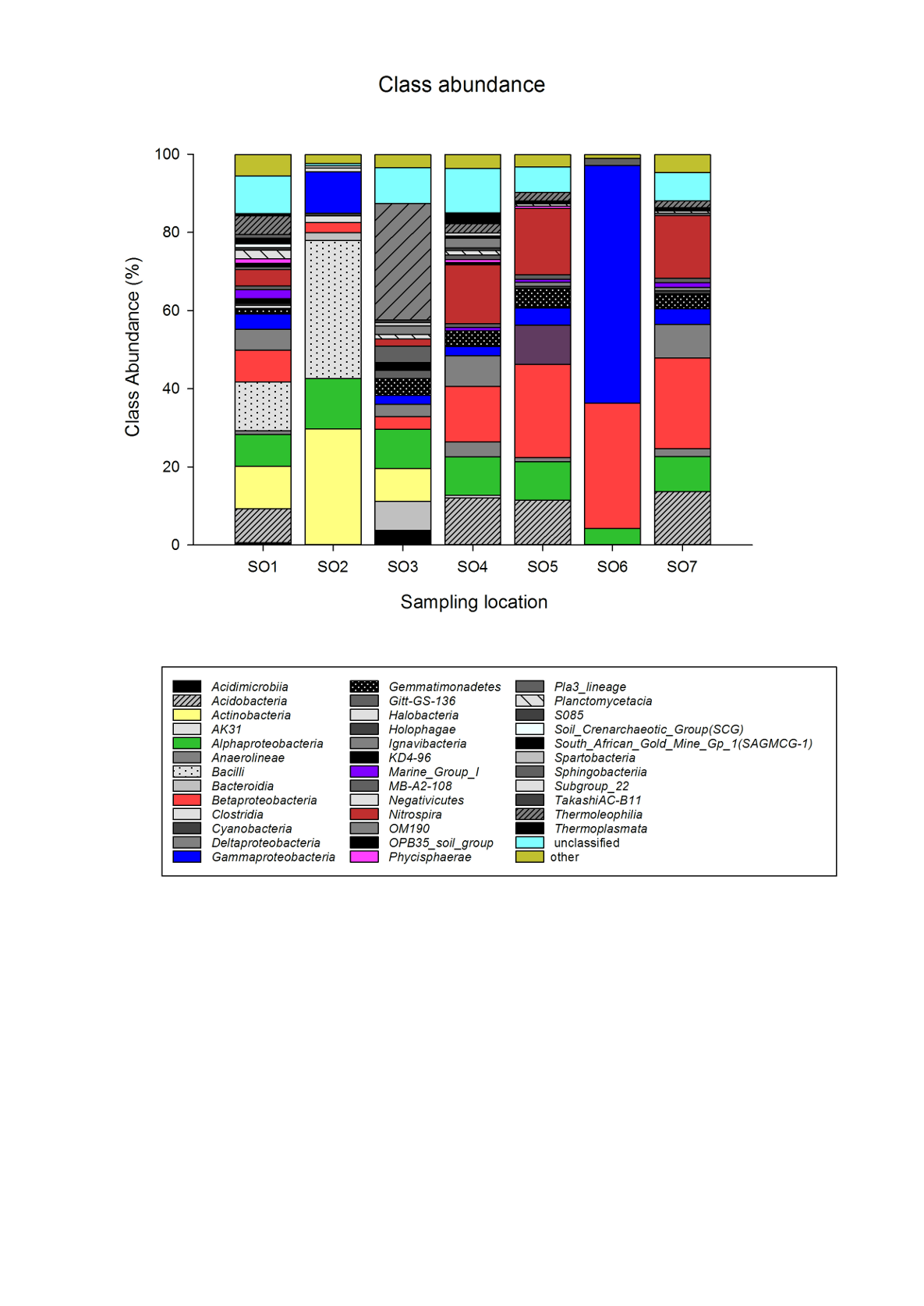

Supplement: S1 Fig — (TIF) [file pone.0180700.s004.TIF]
